# Neural Correlates Underlying Social-Cue-Induced Value Change

**DOI:** 10.1523/JNEUROSCI.2405-21.2022

**Published:** 2022-08-10

**Authors:** Damiano Terenzi, Apoorva R. Madipakkam, Felix Molter, Peter N. C. Mohr, Annabel B. Losecaat Vermeer, Lu Liu, Soyoung Q. Park

**Affiliations:** ^1^Department of Decision Neuroscience and Nutrition, German Institute of Human Nutrition, Potsdam-Rehbrücke, 14558 Germany; ^2^Charité-Universitätsmedizin, Corporate member of Freie Universität Berlin, Humboldt-Universität zu Berlin, and Berlin Institute of Health, Berlin Neuroscience Research Center, 10117 Berlin, Germany; ^3^Deutsches Zentrum für Diabetesforschung, 85764 Neuherberg, Germany; ^4^Department of Psychology, University of Lübeck, 23562 Lübeck, Germany; ^5^School of Business and Economics, Freie Universität Berlin, 14195 Berlin, Germany; ^6^Center for Cognitive Neuroscience, Freie Universität Berlin, 14195 Berlin, Germany; ^7^Wissenschaftszentrum Berlin Social Science Center, D-10785 Berlin, Germany; ^8^Department of Psychology, Sun Yat-sen University, Guangzhou 510631, China

**Keywords:** behavioral modification, fMRI, gaze, social cue, value processing

## Abstract

As humans are social beings, human behavior and cognition are fundamentally shaped by information provided by peers, making human subjective value for rewards prone to be manipulated by perceived social information. Even subtle nonverbal social information, such as others' eye gazes, can influence value assignment, such as food value. In this study, we investigate the neural underpinnings of how gaze cues modify participants' food value (both genders) by means of functional magnetic resonance imaging. During the gaze-cuing task, food items were repeatedly presented either while others looked at them or while they were ignored by others. We determined participants' food values by assessing their willingness to pay before and after a standard gaze-cuing training. Results revealed that participants were willing to pay significantly more for food items that were attended to by others compared with the unattended to food items. Neural data showed that differences in subjective values between the two conditions were accompanied by enhanced activity in the inferior frontal gyrus, middle temporal gyrus, and caudate after food items were attended to. Furthermore, the functional connectivity between the caudate and the angular gyrus precisely predicted the individual differences in the preference shift. Our results unveil the key neural mechanism underlying the influence of social cues on the subjective value of food and highlight the crucial role of social context in shaping subjective value for food rewards in human.

**SIGNIFICANCE STATEMENT** We investigated how social information like others' gaze toward foods affects individuals' food value. We found that individuals more often choose food items that were looked at by another person compared with food items that were ignored. Using neuroimaging, we showed that this increased value for attended to food items was associated with higher brain activity in the inferior frontal gyrus, middle temporal gyrus, and caudate. Furthermore, functional connectivity between the caudate and the angular gyrus was associated with individual differences in values for food items that were attended to by others versus being ignored. These findings provide novel insights into how the brain integrates social information into food value and could suggest possible interventions like using gaze cuing to promote healthier food choices.

## Introduction

Imagine your lunch break with colleagues at an exotic restaurant. When choosing the lunch menu, you might be interested in knowing what your colleagues are attending to. Can such subtle social information influence your food choice? Starting from early life, humans follow the direction of the gaze of another person (Shepherd, 2010), also known as joint attention. This gaze-following response shifts the observer's attention to the gazed-at target. Interestingly, objects that are looked at by another person are processed faster (shorter reaction times) than non-looked-at objects (Sato et al., 2007; Madipakkam et al., 2019). Importantly, also the affective evaluations of objects are influenced by social cues like gaze. When objects are attended to by another person, they are more liked than ignored (Bayliss et al., 2007; Ulloa et al., 2015). This effect seems to be specifically elicited by gaze cues as other cues (e.g., pointing fingers or arrows) did not result in a similar increase in liking evaluations (Bayliss et al., 2007). Therefore, gaze cues might act as social reinforcers by highlighting objects in the environment that are attractive for another person (Shimojo et al., 2003; Bayliss et al., 2007; Terenzi et al., 2021).

Supporting this idea, a study by Madipakkam et al. (2019) has shown that gaze cuing also alters the evaluation of food. Specifically, participants spent more money, revealed by willingness to pay (WTP), for foods that were repeatedly looked at by another person than unattended to foods. Interestingly, this value shift occurred in the absence of participants' conscious awareness because they were not aware of the contingency between the gaze cue and food item presentation. This study suggests that another's attention may implicitly amplify subjective value in decision-making and more generally that social context can influence individuals' food value (Madipakkam et al., 2019). Further, it provides insights for possible interventions. For example, using gaze cuing to influence food value might promote healthier food choices. Understanding the underlying neural underpinnings of this value change will help gain insight into its underlying cognitive mechanisms, which are completely unknown. This adds an important dimension to the behavioral findings by providing insights for possible neurobiological interventions based on either neuromodulation techniques or pharmacology.

Neuroimaging studies have shown that the orbitofrontal cortex (OFC; Plassmann et al., 2007) and the striatum (Knutson et al., 2007; Levy and Glimcher, 2012) have a crucial role in value processing (e.g., WTP for food), whereas brain regions including the dorsolateral prefrontal cortex and the anterior cingulate cortex may play a role in choice execution and choice conflict, respectively (Botvinick et al., 2004; Rangel and Hare, 2010; Terenzi et al., 2022). However, whether and how this neural network plays a role also in value changes driven by a gaze-cuing paradigm is unclear. In particular, the processing of information on the other's gaze direction (Ramezanpour and Thier, 2020), as well as memory-related mechanisms for value assignment may modulate the neural network underlying value computations (Schonberg and Katz, 2020).

In the present work we used functional magnetic resonance imaging (fMRI) to examine the neural mechanisms of value changes attributed to social information by applying the gaze-cuing paradigm. First of all, based on studies showing gaze-cuing-induced value changes (Madipakkam et al., 2019; Terenzi et al., 2021), we hypothesized that participants' WTP for foods that are attended to by others increases compared with that for foods that are ignored. On a neural level, we hypothesized that this increased WTP is associated with higher brain activity in brain regions encoding the subjective value of rewards such as the OFC and the striatum (Plassmann et al., 2007; Rolls, 2021). We expected to observe a change in these brain areas when comparing postevaluations versus pre-evaluations. Further, we explored how these brain regions change their functional connectivity with other brain regions during the encoding of such food value change. Finally, we hypothesized if these brain activations would be sensitive already during the training phase.

## Materials and Methods

### Participants

Twenty-nine participants took part in the study. Two participants had incomplete data because of technical problems during the acquisition, and therefore their data were excluded from all the analyses, resulting in a sample size of 27 participants (19 female, mean age 24.7, SD 4.6). All participants had normal or corrected-to-normal vision. They had no history of neurologic and psychiatric disorders. Written informed consent was obtained from all participants before the study. The study was in accordance with the Declaration of Helsinki and approved by the ethics committee of the University of Lübeck.

### Stimuli

Stimuli were 36 food pictures of Korean snack items that were not familiar to the participants. Face stimuli consisted of four neutral faces (two males) taken from the Radboud Face Database (Langner et al., 2010; Madipakkam et al., 2019). The three versions of each face stimulus were looking straight ahead, looking to the left side, or looking to the right side.

### Design and procedure

**Figure 1.**
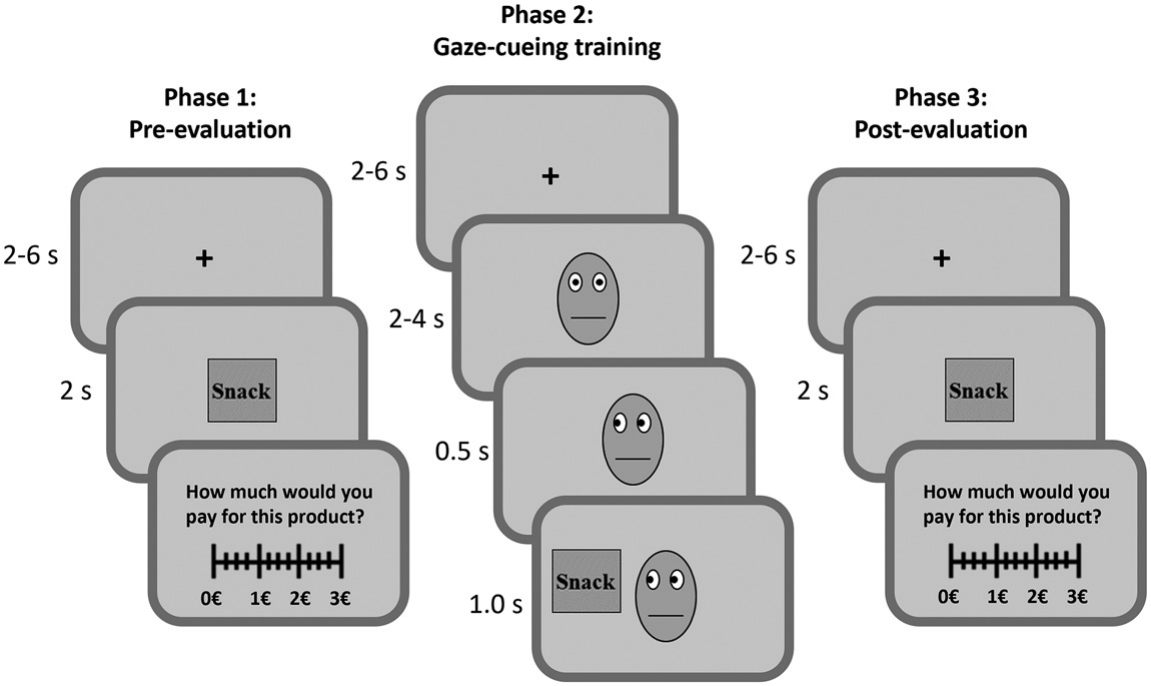
Task design. Middle, Example of a congruent trial sequence in the Gaze-cuing phase. In the pre-evaluation (left) and postevaluation phase (right), participants provided their WTP evaluations for each food item. Changes in WTP (postevaluations vs pre-evaluations) were assessed after the Gaze-cuing phase (center). The face and snack stimuli shown here are stylized representations of the original stimuli.

The experiment consisted of three different phases, pre-evaluation, gaze cuing and postevaluation (Fig. 1). FMRI data were collected during each phase.

The subjects received 10 Euros per hour for participation as well as an additional 3 Euros to buy one of the snack items randomly chosen at the end of the experiment. During the pre-evaluation phase, subjects indicated their WTP for each food item by means of the Becker–DeGroot–Marschak auction (Becker et al., 1964). This procedure allowed us to measure the individual's subjective value of the food items. Choices were made using a scale ranging from 0 to 3 Euros (in 20-cent increments). Each food item was presented three times (108 trials in total). However, only in one third of the trials (36 trials), following the presentation of the foods participants were asked to provide their WTP for the products. Ratings were self-paced.

After the pre-evaluation phase, participants performed the gaze-cuing training phase. Here, the food items were sorted into congruent, incongruent, and neutral conditions (12 food items in each category). Each trial started with a fixation cross (jittered duration between 2 and 6 s) followed by a neutral face looking straight ahead (jittered duration between 2 and 4 s). After that, the face made a gaze shift (0.5 s), either to the left or to the right, or continued to look straight ahead. Finally, a food item was presented on the same side of the gaze shift (congruent trials) or on the opposite side (incongruent trials). In neutral trials, the face continued looking straight ahead while the food item was randomly presented on the right or left side of the face stimulus. Participants were told to respond as quickly and accurately as possible by indicating where the food item was presented (left or right side of the face) via button press. When participants pressed the wrong button, the trial was counted as incorrect. There were six runs of 36 trials each (216 trials in total).

**Figure 2.**
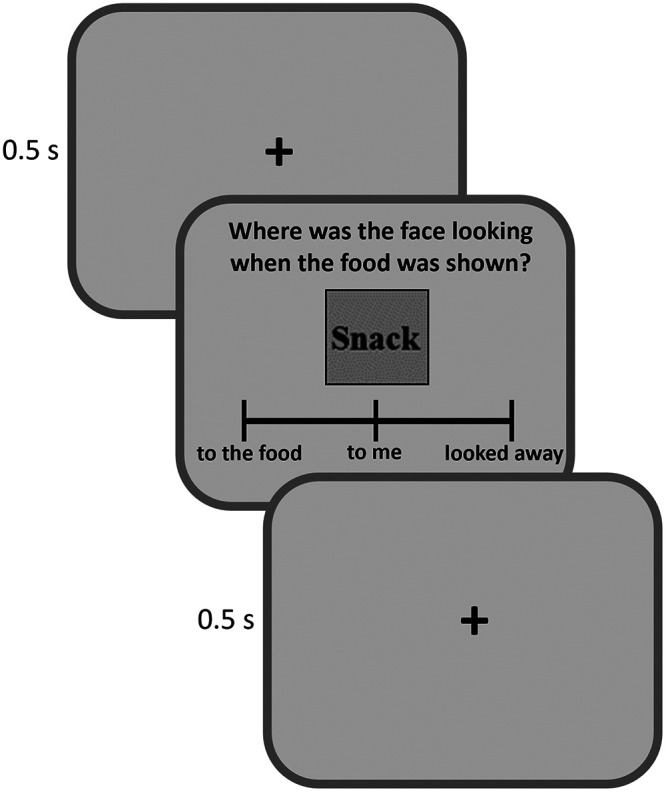
Example of a trial sequence in the discrimination test. During this test each food item was shown on the screen together with the question asking the direction of the gaze of the face cue, with the options to answer “to the food” (congruent condition), “looked away” (incongruent condition), or “to me” (neutral condition). Participants could respond at their own pace using a mouse cursor to indicate their answer. Food items were presented in random order with an ITI of 0.5 s.

After the gaze-cuing training phase, participants' WTP for the same food products was measured again in the postevaluation phase. Finally, participants were asked to perform a discrimination task to test whether they were aware about the contingency between the direction of the gaze and the location of the food item. Moreover, during the discrimination test, each of the 36 food items was shown on the screen together with a question asking the direction of the gaze of the face cue when the same food was presented during the gaze-cuing training; the options to answer were “to the food” (congruent condition), “looked away” (incongruent condition), or “to me” (neutral condition). Participants could respond at their own pace using a mouse cursor to indicate their answer. Food items were presented in random order with an an intertrial interval (ITI) of 0.5 s (Madipakkam et al., 2019; Fig. 2).

### Data analyses

#### Behavioral data

Data were analyzed using R software (https://www.r-project.org/). The Shapiro–Wilk test was undertaken to demonstrate that data were normally distributed. We combined the incongruent and neutral conditions to have all food items that were not looked at in one control condition. This control condition was used in the analyses as no differences emerged between the incongruent and the neutral condition in the change of the WTP ratings after the gaze-cuing training (*t*_(26)_ = 2.09; *p* = 0.139). No differences emerged between these two conditions also in a previous behavioral study using a similar paradigm (Madipakkam et al., 2019). Thus, we report only the results using one single control condition.

To measure the change of the WTP after the gaze-cuing phase, for each food item, pre-evaluations were subtracted from the postevaluations. These scores were then normalized based on the length of the rating scale as follows: 100 * (postevaluations – pre-evaluations)/length of scale. Finally, these normalized values were mean centered. We then entered these values in a paired *t* test between the congruent and the control conditions.

For the gaze-cuing phase, response times and accuracy scores were entered in two separate paired *t* tests for the congruent and control conditions comparisons.

Participants' performance in the discrimination task was assessed using a one-sample *t* test against the chance level of 33% (Madipakkam et al., 2019). Further, a Pearson's correlation analysis was performed between participants' WTP changes in the congruent versus control condition and their performance in the discrimination task. One subject did not perform the discrimination task because of technical problems.

#### Neuroimaging data

Data were acquired with a 3T MRI system (Siemens Magnetom Skyra) using a 64-channel head coil. fMRI data were obtained using an echoplanar imaging sequence with the following parameters (axial slices = 37; interslice gap = 0.6 mm; repetition time (TR) = 2000 ms; echo time (TE) = 30 ms; flip angle = 70°; voxel size = 3.0 × 3.0 × 3.0 mm^3^; field of view (FOV) = 192 × 192 mm). The data were acquired parallel to the commissural line (anterior commissure–posterior commissure) in an interleaved manner. High-resolution structural images were acquired using a 3D sagittal T1-weighted magnetization prepared-rapid gradient echo sequence (sagittal slices = 176; TR = 1900 ms; TE = 2.52 ms; voxel size = 1.0 × 1.0 × 1.0 mm^3^; flip angle = 9°; inversion time (TI) = 900 ms; FOV = 256 × 256 mm^2^).

Spatial processing, subject-level modeling, and group-level statistics were conducted using SPM12 software (Wellcome Trust Center for Neuroimaging) toolbox in MATLAB (version R2019a, MathWorks). The functional imaging data were slice-time corrected, head-motion corrected (Scans with displacement ≥3.0 mm were excluded.), spatially realigned, normalized to the standard Montreal Neurological Institute (MNI) template (voxel size = 3 × 3 × 3 mm^3^), and spatially smoothed with a Gaussian kernel (full-width at half-maximum of 8 mm).

To examine neural responses that related to the gaze-cuing training, we first setup a general lineal model (GLM) with four events (EVs), (1) indicator for congruent trials, (2) indicator for incongruent trials, (3) indicator for neutral trials, and (4) indicator for the face cue. This analysis was performed using food onsets. Further, six movement parameters were included as regressors of no interest. The regressors were convolved with the hemodynamic response function (HRF). To remove low-frequency signal drift, a high-pass filter (128 Hz) was applied. Next, we calculated for each participant the contrasts of congruent > incongruent conditions, congruent > neutral conditions, as well as congruent > control (across incongruent and neutral) conditions, to examine brain activation related to the gaze-cuing training. For each of the first three EVs, congruent, incongruent, and neutral, an additional parametric regressor (i.e., the count, from one to six of each picture across the six runs) was included to further quantitatively assess the modulation effect of picture exposure frequency on brain activation. These contrast images were entered in a second-level random-effects analysis using a voxel-wise one-sample *t* test. When examining the brain imaging data that related to the gaze cuing training phase, four participants were excluded as their functional scans were incomplete because of technical error, resulting in a sample of 23 participants for analyses of the gaze-cuing effects on brain activity.

Next, to examine the neural signature of value change because of the gaze-cuing training (postevaluations greater than pre-evaluations), we first set up a GLM with the following six regressors: three regressors for food bidding (WTP of the three experimental conditions congruent, incongruent, neutral), and an additional three regressors for food-stimulus presentation of the three conditions without WTP (passively looking). Further, six movement parameters were included as regressors of no interest. The regressors were convolved with the HRF. Next, we calculated for each participant the contrast of congruent versus incongruent conditions and congruent versus neutral conditions, as well as congruent versus control conditions. These resulting contrast images were used in a second-level analysis using a voxel-wise paired-sample *t* test. Regarding the pre- and postevaluation phases, the functional images of two participants were excluded as they were incomplete because of technical error. Thus, these neuroimaging analyses included 25 participants.

To test the hypothesized gaze-cuing training effect in reward related regions to food stimuli (for the contrast, congruent greater than control), a priori defined region of interest (ROI) analyses in the striatum and the OFC were performed. ROIs were defined using bilateral masks of the OFC, as well as bilateral masks of the striatum including putamen, caudate nucleus, and ventral striatum. These ROIs were derived from the Harvard-Oxford atlas (http://fsl.fmrib.ox.ac.uk/fsl/fslwiki/Atlases; we applied the Harvard-Oxford atlas to MNI space with the DAPBI toolbox).

Next, ROIs that showed significant brain activations, as well as significant activations that emerged from whole-brain analyses, were entered in different analyses using the generalized psychophysiological interactions (gPPI) toolbox (https://www.nitrc.org/projects/gppi) to compute the functional connectivity at the time of the WTP evaluations (contrast, congruent vs control) and its association with WTP changes (postevaluations vs pre-evaluations; McLaren et al., 2012).

Further, to test whether voxels emerged from the contrast (congruent greater than incongruent) were the same as those identified in the contrast (congruent greater than neutral), a conjunction analysis was performed using the xjView toolbox (https://www.alivelearn.net/xjview). Whole-brain statistical maps were voxel-level thresholded at *p* < 0.001 before undergoing cluster-level familywise error (FWE) correction (*P_FWE_* < 0.05). We report significant brain activations within the ROIs that survived FWE correction for multiple comparisons using small-volume correction (*P_SVC-FWE_* < 0.05).

## Results

We conducted a within-subject experiment with three different phases, pre-evaluation, gaze-cuing training, and postevaluation **(**Fig. 1; see above, Materials and Methods). The pre- and postevaluation phases were identical. Here, subjects submitted their WTP for 36 different Korean food items, which were previously unfamiliar to them. Choices were made using a scale ranging from 0 to 3 euros (in 20-cent increments). Between the pre- and postevaluations, participants underwent a gaze-cuing training in which the food items were repeatedly presented together with a face. Some food items were repeatedly looked at by others (congruent condition), whereas others were ignored by others (control condition). Participants were instructed to respond with a button press as quickly and accurately as possible by indicating where the food item was presented (left or right side of the central face cue) via button press. Finally, participants were asked to also perform a discrimination task to test whether they were aware about the contingency between the direction of the gaze and the location of the food item during the gaze-cuing training (see above, Materials and Methods).

### Behavioral results

#### Looked-at food items are attended to faster (gaze-cuing training phase)

**Figure 3.**
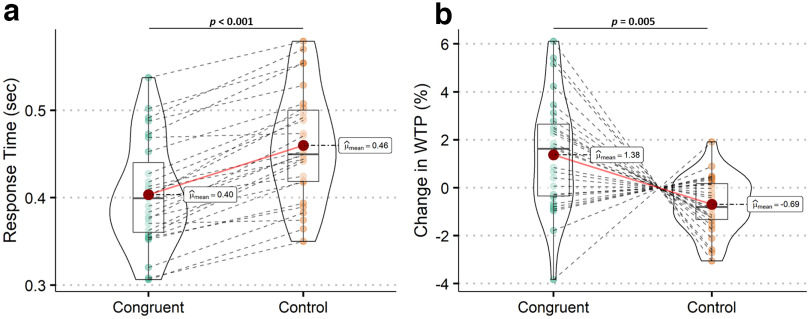
Results from the gaze-cuing training and the evaluation phases. ***a***, Participants respond faster when food items are looked at compared with being ignored (*t*_(26)_ = −11.6; *p* < 0.001), during the gaze-cuing training. ***b***, When comparing postevaluations versus pre-evaluations, WTP (%) significantly increased for the looked-at food items compared with the ignored items (*t*_(26)_ = 3.04; *p* = 0.005).

We first investigated whether the gaze-cuing training facilitated participants' response when the food items appeared on the screen (to the left or the right of the central cue). In particular, we tested the hypothesis that the time to detect targets that were looked at by others (congruent condition) was shorter than that for non-looked-at targets (control condition) because of gaze-evoked shifts in participants' attention. To do so, we compared participants' response time (button presses) in these two conditions. A paired *t* test showed that participants were faster in responding to the looked-at food items (congruent condition) compared with the non-looked-at food items (control condition; *t*_(26)_ = −11.6; *p* < 0.001; Fig. 3*a*).

Further, participants performed the gaze-cuing training with high accuracy (M = 97%, SD = 0.03). A paired *t* test showed that participants performed similarly well in both the congruent and the control condition (*t*_(26)_ = 1.05, *p* = 0.30).

#### Looked-at food items are preferred more (postevaluation vs pre-evaluation phases)

Next, we checked whether the gaze-cuing training has an impact on the food value by comparing the food WTP of postevaluation versus pre-evaluation phases (see above, Materials and Methods). In particular, we hypothesized that participants' WTP for attended to foods increased compared with ignored foods. A paired *t* test on subjects' WTP changes showed that participants were willing to spend more money for the looked-at items compared with the control items (*t*_(26)_ = 3.04, *p* = 0.005; Fig. 3*b*). Because participants were asked to rate different unfamiliar snack foods, we tested through correlation analyses whether their WTP ratings were consistent across the pre- and postevaluation phases. Results show that WTP ratings between pre- and postevaluations in the congruent condition were significantly correlated (*r* = 0.70, *p* < 0.001). Similarly, WTP ratings between pre- and postevaluations for the control condition were significantly correlated (*r* = 0.83, *p* < 0.001). These results suggest that the intrinsic values of the unfamiliar food items used in our study were not arbitrary.

Further, we tested through a discrimination task whether participants were aware of the contingency between the position of the food item and the direction of the gaze during the gaze-cuing phase. A one-sample *t* test (against the chance level of 33%; Madipakkam et al., 2019) of participants' performance in the discrimination task did not show a significant difference from chance (*t*_(25)_ = −0.08, *p* = 0.93). Also, there was no significant correlation between the difference in the change in WTP ratings for congruent versus control items and the participants' performance in the discrimination task (*r* = −0.32, *p* = 0.11), suggesting that the gaze-cue-induced value change occurred unconsciously.

To test for possible sex differences in the change in WTP ratings, we performed a repeated-measures ANOVA on participants' WTP changes with Condition (Congruent, Control) as a within-subjects variable and Sex (Female, Male) as a between subjects. Results did not show a main effect of Sex (*F*_(1,25)_ = 0.07, *p* = 0.793) nor an interaction Condition times Sex (*F*_(1,25)_ = 0.07, *p* = 0.793). Similarly, a repeated-measures ANOVA on RT values did not show a main effect of Sex (*F*_(1,25)_ = 0.015, *p* = 0.905) nor an interaction Condition times Sex (*F*_(1,25)_ = 0.013, *p* = 0.911)

### Neuroimaging results

#### Changes in food value are reflected in increases in middle temporal gyrus, inferior frontal gyrus, and caudate responses (postevaluation vs pre-evaluation phases)

**Figure 4.**
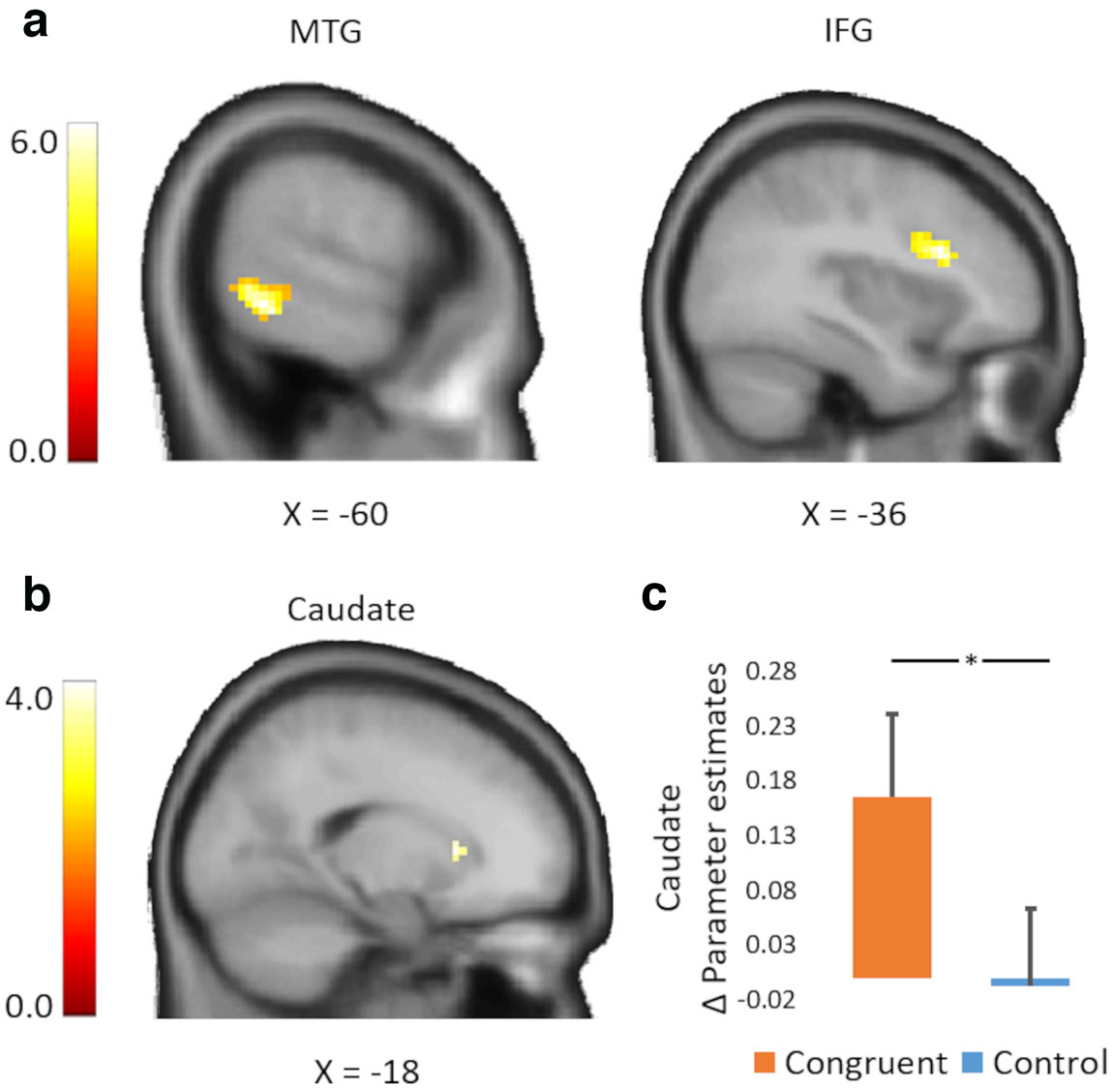
Brain activations related to value changes. ***a***, Whole-brain results showing condition differences (congruent greater than control condition) for WTP ratings (postevaluations greater than pre-evaluations). Greater activations were found in the left MTG and in the left IFG (*P_FWE_* < 0.05). ***b***, Region of interest (ROI) results showing condition differences (congruent greater than control condition) for WTP evaluations (postevaluations greater than pre-evaluations). Greater activations were found in the left caudate (*P*_SVC-FWE_ < 0.05). ***c***, Parameter estimates extracted from the caudate region that showed significant condition differences. The asterisk (*) indicates *p* <0.05. Error bars indicate SEM.

To examine the neural signature of value change because of the gaze-cuing training (postevaluations greater than pre-evaluations), we compared the neural activation toward food items that were repeatedly looked at (congruent items) in comparison to those that were ignored by others (control items) at the time of WTP evaluations. Whole-brain statistical maps were voxel-level thresholded at *p* < 0.001 before undergoing cluster-level familywise error correction (*P_FWE_* < 0.05). The paired *t* test revealed a greater activation (contrast, congruent greater than control) in the left middle temporal gyrus [MTG; Montreal Neurologic Institute (MNI) coordinates, *x* = −60, *y* = −48, *z* = 9; *t* = 6.59; *P_FWE_* < 0.05, cluster level corrected] and in the left inferior frontal gyrus (IFG; MNI coordinates, *x* = 36, *y* = 18, *z* = 24; *t* = 4.41; *P_FWE_* < 0.05, cluster level corrected) in the postevaluation phase compared with that in the pre-evaluation phase (Fig. 4*a*, Table 1).

**Table 1.** Regions showing condition differences (congruent vs control, congruent vs incongruent, congruent vs neutral) for WTP evaluations (postevaluation vs pre-evaluation)

			Peak MNI				
	Hemisphere	Cluster size	*x*	*y*	*z*	Peak *t* value	*p* Value cluster (uncorrected)
Brain regions (congruent vs control)							
Middle temporal gyrus	Left	114	−60,	−48	9	6.59	*p* = 0.001
Inferior frontal Gyrus	Left	160	−36	18	24	4.41	*p* < 0.001
Brain regions (congruent vs incongruent)							
Inferior frontal gyrus	Left	149	−57	15	30	5.03	*p* < 0.001
Parietal lobe/precuneus	Left	97	−30	−63	24	4.59	*p* = 0.003
Brain regions (congruent vs neutral)							
Superior frontal gyrus	Left and Right	80	−9	30	60	3.95	*p* < 0.001

*P*_FWE_ < 0.05.

**Figure 5.**
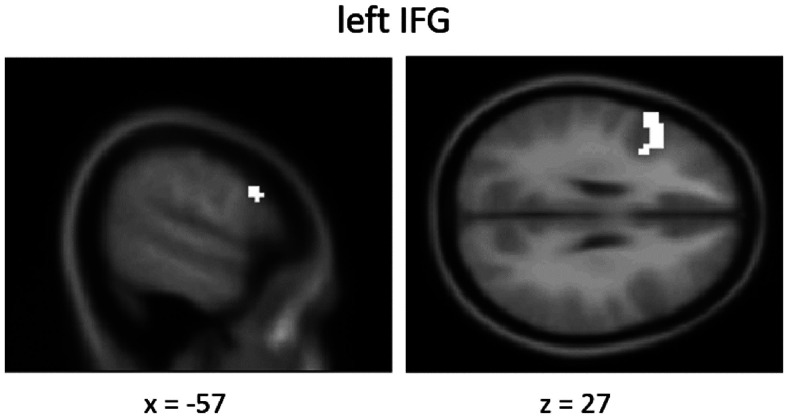
Conjunction analysis. The conjunction analysis shows an area of overlap (postevaluations vs pre-evaluations) for the two contrasts congruent versus incongruent and congruent versus neutral within the left inferior frontal gyrus (*p* < 0.005, uncorrected; *k* ≥ 10).

**Figure 6.**
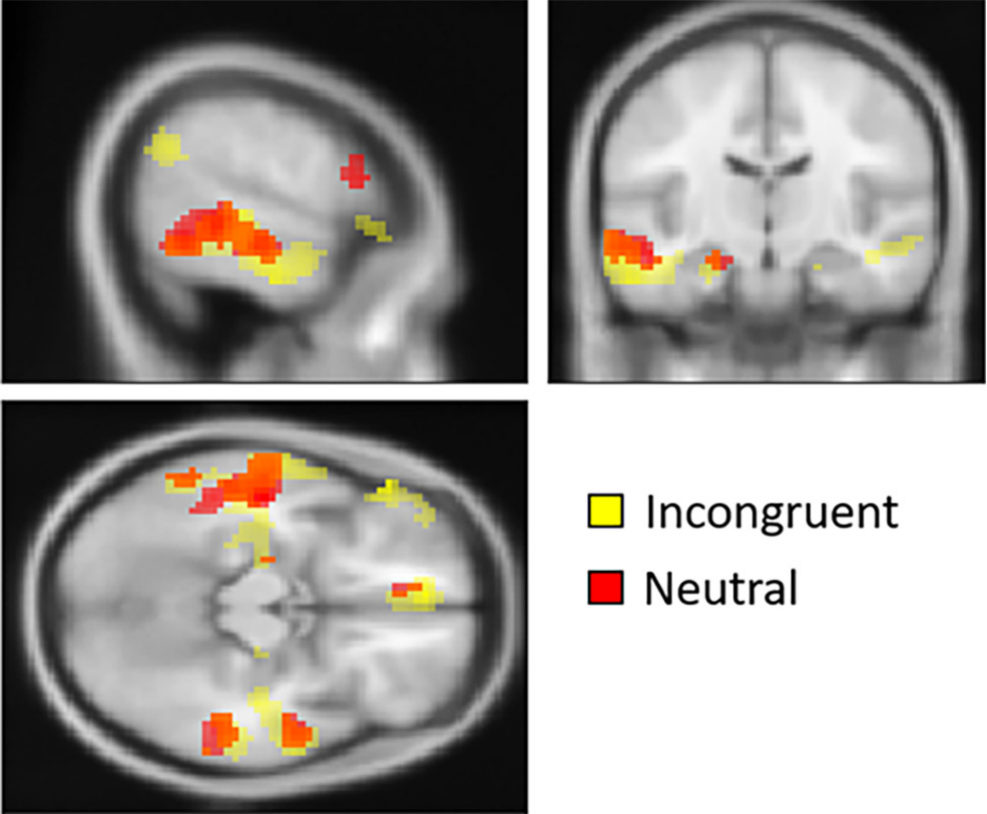
Conjunction analysis. The conjunction analysis shows areas of overlap in the left and right MTG, left Inferior Temporal Gyrus (ITG), and left angular gyrus for the incongruent (in yellow) and neutral (in red) conditions (postevaluations vs pre-evaluations; *p* < 0.005, uncorrected; *k* ≥ 10).

We then focused our analyses on the brain regions that were shown to code reward value (Knutson et al., 2007; Rangel et al., 2008; Levy and Glimcher, 2012) by means of a priori defined ROI analyses in OFC and the striatum (see above Materials and Methods). These analyses revealed greater blood oxygenation level-dependent (BOLD) activity (contrast, congruent greater than control) in the left caudate in the postevaluation phase compared with the pre-evaluation phase (cluster size = 149 voxels, *t* value = 3.57; cluster corrected *p* = 0.022) during the WTP ratings (Fig. 4*b*). No other significant results emerged. Table 1 and Figures 5 and 6 show the analyses including all three conditions (congruent, incongruent, and neutral).

**Figure 7.**
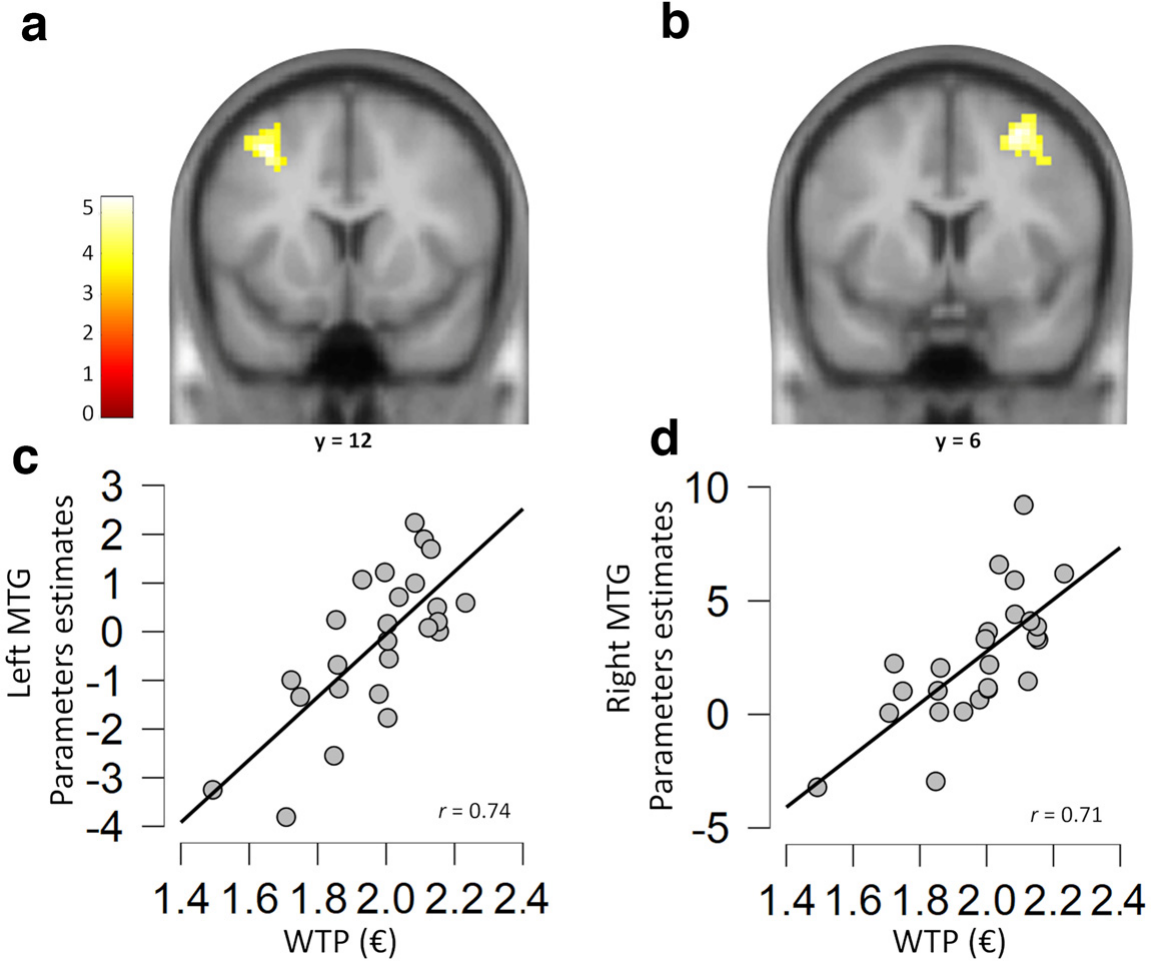
Results from the multiple regression analysis. ***a***, ***b***, BOLD activity in the left MFG (***a***) and right MFG (***b***) at the time of the food presentation in the pretest phase significantly predicted WTP ratings. Specifically, across participants, higher activity in these brain regions was associated with greater WTP for unknown food items. ***c***, ***d***, Scatter plots are for the purpose of data visualization only.

We further examined brain activations related to the pretest phase at the time of the food presentation (using food onsets) and their associations with WTP ratings (one averaged WTP value across the different conditions for each participant). A multiple regression analysis was used to test for associations between WTP ratings and the BOLD responses at the time of food presentation. Results revealed activation in the left middle frontal gyrus (MFG; MNI coordinates, *x* = −33, *y* = 12, *z* = 45; *t* = 5.37; *P_FWE_* < 0.05, cluster level corrected) and right MFG (MNI coordinates, *x* = 30, *y* = 6, *z* = 54; *t* = 5.07; *P_FWE_* < 0.05, cluster level corrected), which were positively associated with WTP ratings (Fig. 7). To determine the association between the BOLD responses in these brain regions (6 mm sphere centered on the peak clusters identified) and WTP ratings, we performed correlational analyses. Results revealed that higher activities in these brain regions were associated with greater WTP for food items, meaning that these areas precisely reflected the food evaluations (Fig. 7). These results suggest that higher activity in the bilateral MFG precisely reflected greater WTP ratings for unknown food items. In addition, we examined whether the caudate is involved in the evaluation of foods in both the pre-evaluation and postevaluation phases. Results revealed significant activations in the left caudate at the time of the food presentation in the pre-evaluation phase (cluster size = 145 voxels, peak *t* value = 3.00; cluster corrected *p* = 0.021), as well as in the postevaluation phase (cluster size = 138 voxels, peak *t* value = 1.25; cluster corrected *p* = 0.023), with using a predefined caudate ROI. These results suggest that the caudate activity not only reflects the change of WTP but also the value representation itself. No other significant results emerged when using other predefined ROIs (bilateral masks of the OFC, putamen, and ventral striatum) for both pre- and postevaluation phases

#### Caudate-connectivity modulation with angular gyrus reflects gaze-cue-induced value change

Next, we aimed to understand the connectivity changes of the brain regions that showed a change as a function of the training to understand how they interact with other brain regions. To do so, we examined whether ROIs that showed significant brain activations, as well as significant activations that emerged from the whole brain analyses may interact with other brain regions. In particular, we aimed to examine whole-brain functional connectivity of (1) the clusters of left IFG and MTG and (2) the entire left caudate ROI for the contrast postevaluation (congruent vs control) versus pre-evaluations (congruent vs control). To do so, we performed different gPPI analyses (see above, Materials and Methods).

**Figure 8.**
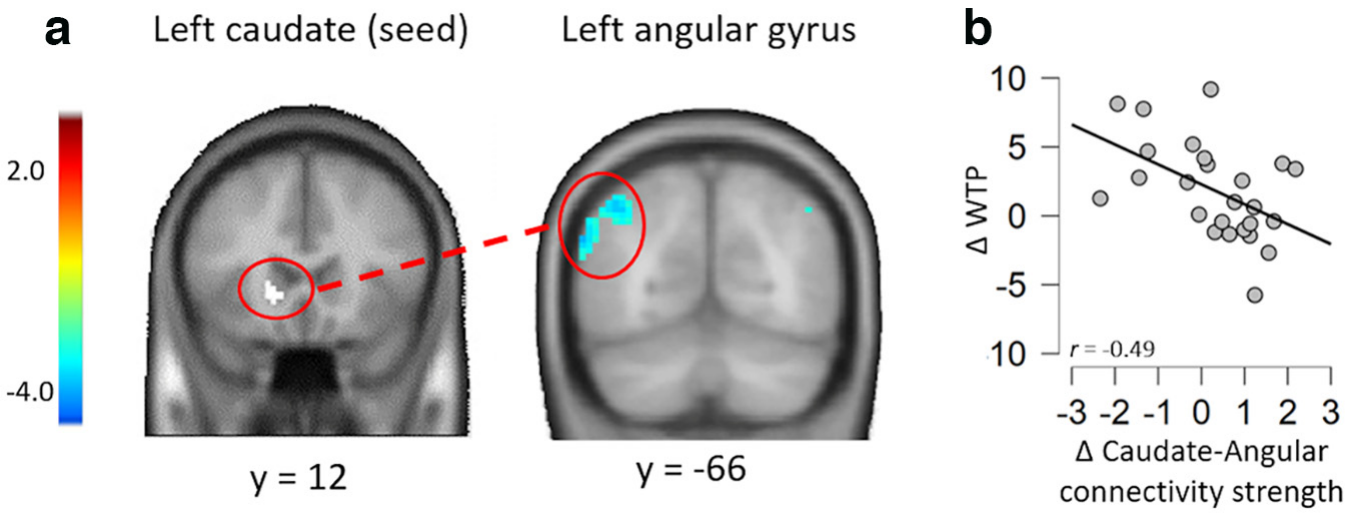
Results from the seed-based ROI caudate gPPI functional connectivity analysis. ***a***, Caudate-angular connectivity change during congruent versus control trials significantly predicted WTP changes (postevaluations vs pre-evaluations; cluster size, 176 voxels, *t* value = −2.82, *P_FWE_* = 0.024). ***b***, Specifically, the correlation between caudate-angular connectivity and WTP changes were negative. In other words, across participants, the smaller the connectivity change the greater WTP change was observed. Scatter plot is for the purpose of data visualization only.

The results of the gPPI analyses (initial voxel level at *p* < 0.005 for cluster formation and then cluster-level familywise error corrected, *P_FWE_* < 0.05) revealed a significant left caudate seed-angular gyrus connectivity that was significantly associated with the WTP change (cluster size = 176 voxels, *t* value = −2.82, *P_FWE_* = 0.024; Fig. 8*a*). To note, this result did not survive when applying a more stringent criterion (e.g., voxel-level *p* < 0.001, *P_FWE_* < 0.05). To determine the association between the caudate-angular gyrus connectivity and the change in WTP ratings, we conducted a correlation analysis between the connectivity changes extracted from the angular gyrus cluster (6 mm sphere centered on the peak angular gyrus cluster identified in the contrast congruent greater than control) and WTP changes. We observed that a lower left caudate angular gyrus connectivity during congruent versus control trials (in the postevaluation vs pre-evaluation phases) was associated with a greater WTP change for food items (Fig. 8*b*). Last, to examine a possible association between the change in the left caudate individually (6 mm sphere centered on the peak caudate cluster identified for the contrast congruent greater than control) and the change in WTP, a Pearson's correlation was performed. Results showed no significant correlation (*r* = 0.03, *p* = 0.89). There was no activation change in the left angular gyrus at the whole-brain level (*P_FWE_* < 0.05).

#### Gaze-cue training phase

Next, we examined brain activations related to the gaze-cuing training and compared the BOLD activity toward congruent items in comparison to that for control items during the training phase (see above, Materials and Methods). Whole-brain statistical maps were voxel-level thresholded at *p* < 0.001 before undergoing cluster-level familywise error correction (*P_FWE_* < 0.05). The one-sample *t* test revealed no significant results for this contrast.

Further, we also tested activation change within the striatum and OFC as a priori independent ROIs. These analyses revealed greater BOLD activity (contrast, congruent greater than control) in the left caudate (cluster size = 149 voxels, *t* value = 1.08; cluster corrected *p* = 0.020). No other significant results emerged.

## Discussion

By combining fMRI and a gaze-cuing paradigm, we investigated the neural underpinnings of individuals' food value changes modulated by social information.

First, we show that the value of food that is attended to by others increases compared with ignored food, a replication of the previous finding (Madipakkam et al., 2019). Particularly, we found that participants were willing to pay more money for food items attended to by others compared with ignored ones. This finding corroborates the role of a powerful social cue like gaze not only in shifting the observers' attention but also in altering their subjective value for food rewards. Our results may be particularly relevant to understand the establishment of food preferences, consumer behavior, and food choice in early life. For example, it has been shown that gaze cues facilitate object processing in 4-month-old children (Wahl et al., 2019). Further, children are more prone to eating new foods when an adult model also eats them, compared with eating alone (Harper and Sanders, 1975).

Strikingly, in our study, we found that this change can even occur implicitly as participants were not aware of the contingency between the gaze cue direction and the position of the food item. This result suggests that individuals may use gaze to rapidly establish preferences about stimuli in the social environment, an ability that might be the result of an evolutionary adaptation because it allows to orient the attention to discoveries made by others (Shepherd, 2010).

Second, we found that participants were faster in detecting targets that were gazed at by others than that for non-gazed-at targets, which is in line with a large number of studies using gaze-cuing paradigms. This observation confirms that gaze is a powerful cue for attention (Atkinson et al., 2018).

On a neural level, we found that the increased WTP for food items previously presented in congruent trials during the gaze-cuing training phase (compared with the WTP for items presented in control trials) was reflected in increased activity in the left IFG (pars opercularis and triangularis), left caudate, and the left MTG. Interestingly, in line with our results, previous studies have reported the involvement of the left IFG in attentional orienting to eye gaze cues (Bayless et al., 2013; Atkinson et al., 2018) during reward responsiveness (Rademacher et al., 2010; Liu et al., 2011), as well as during the processing of the subjective value of rewards in decision-making tasks (Massar et al., 2015; Castrellon et al., 2019). Particularly, a study by Castrellon et al. (2019) has found a positive correlation between mesolimbic dopamine D2-like receptors in the ventral striatum and the activity of the left IFG during subjective value computations (Castrellon et al., 2019). Similarly, we found increased activity in the left striatum and the left IFG in the postevaluation phase compared with the pre-evaluation phase for food items previously presented in congruent trials (vs control trials) during the gaze-cuing phase. Evidence for value coding not only in the ventral striatum but also in the caudate region has been found in other previous studies (Delgado et al., 2004; Tricomi and Lempert, 2015; Schultz, 2016). Particularly, it has been proposed that similar to the left ventral striatum, the left dorsal striatum is involved in value coding, whereas the right dorsal striatum is involved in probability coding (Tricomi and Lempert, 2015). In our study, although the ventral striatum showed no significant activation, the dorsal part (caudate) reflected value processing. However, no significant functional connectivity was found between the left caudate and the left IFG. It may be possible that the different task used in our study results in different neural correlates. Indeed, in the study by Castrellon et al. (2019), the subjective value of rewards was measured through a delay discounting task, a paradigm particularly used to measure impulsive decision-making (Kable and Glimcher, 2007; Terenzi et al., 2019); whereas in our study, participants' subjective value of rewards was measured through WTP ratings before and after a gaze-cuing task. It should be noted that we used predefined ROIs (including the OFC and striatum) based on previous studies investigating subjective value. However, these ROIs (despite being abundant in the literature) may be the result of the signal-to-noise ratio; thus, there is the need for caution when interpretating the functioning of these brain areas in computing subjective value (Poldrack, 2007; Elliott et al., 2020).

Interestingly, results emerging in our study show a left-hemispheric dominance reflecting the increased subjective value of food items attended to by others. This left lateralization has also been reported in other studies investigating approach motivation (Pizzagalli et al., 2005) and consumer choices (Ohme et al., 2009, 2010; Mengotti et al., 2018). However, other studies have shown mixed results (e.g., stronger right-lateralized brain areas supporting reward-related behaviors), thus suggesting that additional research is still needed to understand a possible hemispheric asymmetry in the calculation of the subjective value of rewards (Plassmann et al., 2007; Tricomi and Lempert, 2015; Ramsøy et al., 2018).

Another neural signature of value changes because of the gaze-cuing training (postevaluations greater than pre-evaluations) found in this study is the increased activity in the left MTG. Previous studies have reported the involvement of this brain region in different functions such as semantic memory processing and visual perception (including spatial attention and gaze processing; Lockhofen et al., 2014; Ren et al., 2020). It might be possible that this area played a role in retrieving spatial memories associated with food items previously presented in different conditions during the gaze-cuing phase, thus influencing the calculation of the subjective value of the reward.

A further interesting neural signature of value change observed in this study is the functional caudate-angular connectivity and its modulation by WTP changes. In particular, across participants, the caudate angular gyrus connectivity decreased with increasing WTP changes. Further, when correlating the individual activation change in the left caudate with the WTP change, there was no significant correlation. This result suggests that neither the single contribution of the caudate nor the angular gyrus but their functional coupling is involved in the gaze-cue-induced value change. As mentioned earlier, the caudate has been reported to be involved in the processing of the subjective value of rewards (Delgado et al., 2004; Schultz, 2016), whereas the angular gyrus has a role in spatial attention and orientation toward salient stimuli (Seghier, 2013). Thus, it is likely that in the postevaluation phase (compared with the pre-evaluation phase) the connectivity between these two brain areas may be reduced because of facilitation by the gaze-cuing training in the processing of the value for food items looked at by others. This is in line with the hypothesis that the gaze-following response is the result of an evolutionary adaptation, particularly during foraging as it allows one to orient the attention to discoveries made by others (Tomasello and Carpenter, 2007; Zuberbühler, 2008; Shepherd, 2010). However, it should be noted that the caudate–angular gyrus connectivity might indicate that the activity patterns in these brain areas were opposite to each other and not a mere result from facilitation because of the gaze-cue training. Indeed, it has been suggested that although greater functional connectivity might mediate the integration of information between different brain areas, smaller connectivity might dissociate neuronal processes mediating different goals (Fox et al., 2005; Wagner et al., 2020). Thus, the negative interaction between the caudate and the angular gyrus (which was modulated by the increased change in WTP for looked-at food items) might suggest increased value processes during the test phase, potentially segregating a brain region encoding value for rewards (such as the caudate) from activations centered around the angular gyrus that might code for spatial attention. Future research is needed to confirm this finding. Further, our results suggest that the caudate activity not only reflects the change of WTP but also the value representation itself (Delgado et al., 2004; Tricomi and Lempert, 2015; Schultz, 2016). Another brain region emerged in our study and involved in value representation is the MFG. It may be possible that this area was particularly involved in the processing of the logos of the snacks (Bruce et al., 2013), as the food items were presented in their packaging. Because we used unfamiliar food items in our task (Korean snacks), future research is needed to replicate our findings by using other packaging information.

Interestingly, regarding the neural correlates of the gaze-cuing training per se, we found also during this phase an increased activity in the a priori caudate ROI for food items presented in congruent trials (compared with that for items presented in control trials). This result suggests that the left caudate was already sensitive in processing the value of gazed-at food items during the gaze-cuing training. However, we did not find significant activations in MTG, IFG, or any other brain region during this phase.

In conclusion, the present study shows that a gaze-cuing training can modify the value of food as shown both by shift in preference and by orchestrated brain activity and striatal-angular connectivity changes in healthy humans. Our results not only strongly propose novel potential intervention strategy by providing insights into social cue affecting decision processes but also provide evidence of how using gaze-cuing to influence food value can be applied as a powerful tool to actively promote healthy food choices.
